# The clinical efficacy and safety of different biliary drainage in malignant obstructive jaundice: a meta-analysis

**DOI:** 10.3389/fonc.2024.1370383

**Published:** 2024-04-09

**Authors:** Yanzhao Wang, Xuebo Zhao, Yan She, Qian Kang, Xianxia Chen

**Affiliations:** ^1^ Graduate School of Qinghai University, Xining, China; ^2^ Department of Ultrasound Medicine, Qinghai Provincial People’s Hospital, Xining, China

**Keywords:** percutaneous transhepatic cholangial drainage (PTCD), endoscopic retrograde cholangiopancreatography (ERCP), malignant obstructive jaundice (MOJ), meta-analysis, systematic review

## Abstract

**Background:**

Currently, percutaneous transhepatic cholangial drainage (PTCD) and endoscopic retrograde cholangiopancreatography (ERCP) are commonly employed in clinical practice to alleviate malignant obstructive jaundice (MOJ). Nevertheless, there lacks a consensus regarding the superiority of either method in terms of efficacy and safety.

**Aim:**

To conduct a systematic evaluation of the effectiveness and safety of PTCD and ERCP in treating MOJ, and to compare the therapeutic outcomes and safety profiles of these two procedures.

**Methods:**

CNKI, VIP, Wanfang, CBM, PubMed, Web of Science, Embase, The Cochrane Library, and other databases were searched for randomized controlled trials (RCTs) on the use of PTCD or ERCP for MOJ. The search period was from the establishment of the databases to July 2023. After quality assessment and data extraction from the included studies, Meta-analysis was performed using RevMan5.3 software.

**Results:**

A total of 21 RCTs involving 1,693 patients were included. Meta-analysis revealed that there was no significant difference in the surgical success rate between the two groups for patients with low biliary obstruction (P=0.81). For patients with high biliary obstruction, the surgical success rate of the PTCD group was higher than that of the ERCP group (P < 0.0001), and the overall surgical success rate of the PTCD group was also higher than that of the ERCP group (P = 0.008). For patients with low biliary obstruction, the rate of jaundice relief (P < 0.00001) and the clinical efficacy (P = 0.0005) were better in the ERCP group, while for patients with high biliary obstruction, the rate of jaundice relief (P < 0.00001) and the clinical efficacy (P = 0.003) were better in the PTCD group. There was no significant difference in the overall jaundice remission rate and clinical efficacy between the two groups (P = 0.77, 0.53). There was no significant difference in the reduction of ALT, TBIL, and DBIL before and after surgery and the incidence of postoperative complications between the two groups (P > 0.05).

**Conclusion:**

Both PTCD and ERCP can efficiently alleviate biliary obstruction and enhance liver function. ERCP is effective in treating low biliary obstruction, while PTCD is more advantageous in treating high biliary obstruction.

## 1 Introduction

Malignant obstructive jaundice (MOJ) is a prevalent jaundice disorder in hepatobiliary surgery and gastroenterology. It is primarily attributed to the compression of malignant tumor cells (e.g., cholangiocarcinoma, pancreatic head carcinoma, ampullary cancer, etc.) on the relevant tissues of the patient, leading to constriction or even blockage of the bile ducts, bile stasis, and elevated bilirubin. Clinical manifestations encompass skin and scleral yellowing, pruritus, and clay-colored stools. With the rising incidence of biliary cancer, it poses a substantial adverse impact on the physical and mental well-being and daily activities of patients (1, 2).In clinical practice, obstructions from diverse sources can be classified as high and low biliary obstruction, with the intersection of the common hepatic duct and the cystic duct serving as the demarcation point. Hilar tumors typically give rise to high obstructions, while ampullary and peripancreatic tumors commonly result in low obstructions (3).

Currently, the most effective treatment for this disease is surgical resection. Malignant obstructive jaundice caused by malignant tumors of the biliary tract or metastatic carcinomas of the hepatic hilum, particularly biliary cancer jaundice, often lacks specific clinical manifestations in the early stages. Consequently, most patients have missed the opportunity for surgery by the time MOJ emerges (4). With the advancement of interventional therapy techniques, palliative interventional therapy has emerged as the most effective approach to alleviate MOJ. It can effectively reduce the bilirubin level in the blood, safeguard liver function, alleviate jaundice, and enhance the quality of life (5). Percutaneous transhepatic cholangial drainage (PTCD) and endoscopic retrograde cholangiopancreatography (ERCP) form the foundation of palliative intervention (6). PTCD entails the insertion of a percutaneous puncture needle into the intrahepatic bile ducts, followed by the injection of contrast material to visualize the intrahepatic and extrahepatic bile ducts, and subsequent biliary drainage (7). ERCP involves the placement of an endoscope through the patient’s mouth and esophagus into the descending part of the duodenum to locate the opening of the bile ducts and insert a drainage tube. This tube passes through the duodenal papilla to enter the bile ducts for drainage (8).

Currently, there is no consensus regarding the effectiveness of these two treatment options. Domestic and international scholars have systematically assessed the efficacy of PTCD and ERCP in the treatment of MOJ applications, but specific analyses of the efficacy and safety based on the site of obstruction have not been conducted. Therefore, the study aims to compare the clinical efficacy and surgical safety of the two drainage methods, PTCD and ERCP, in the treatment of patients with MOJ with different sites of obstruction, in order to provide a medical basis for clinical treatment.

## 2 Methods

### 2.1 Literature search

Literature search was carried out in two ways: computerized search of articles published in Chinese and foreign language databases (CNKI, VIP, WanFang, CBM, PubMed, Web of Science, Embase, The Cochrane library, etc.). The search strategy is presented in **Appendix Table 1**, and the search period ranges from the establishment of the library to December 2023. Literature was screened based on inclusion and exclusion criteria. Subsequently, similar meta-analyses published in the aforementioned databases were sought, and the full text of the included literature was reviewed to determine whether it met the inclusion criteria of this study. If so, it was included.

### 2.2 Study selection

#### 2.2.1 Inclusion criteria

① Population: Patients diagnosed with MOJ caused by malignancy through pathological and imaging examinations; ② Intervention: PTCD or ERCP; ③ Study Design: Randomized controlled trial ;④ The study clearly reported one or more of the following outcome measures: Surgical success rate, jaundice remission rate, clinical efficacy, liver function index (ALT, TBIL, DBIL), and Complications.

#### 2.2.2 Exclusion criteria

① Repeatedly published literature; ② Non-RCT, meta-analyses, reviews, and animal experiments, etc.; ③ Relevant information is incomplete, unclear, unable to extract valid information, and the study design is unreasonable; ④ Literature without relevant outcome indicators.

### 2.3 Data extraction

Data on authors, year of publication, number of cases, methodological characteristics, and relevant outcome indicators: (1) Surgical success rate: Successful surgery was defined as successful biliary drainage and relief of biliary obstruction. (2) Jaundice remission rate: Five days after the operation, TBIL decreased by more than one-third, indicating the remission of jaundice. (3) Clinical effectiveness: Clinical effectiveness was determined by the reduction of TBIL before and after the surgery. The criteria were as follows: Significant effect: There was a significant improvement in jaundice, and TBIL decreased by more than 30% on the fifth day after the operation. Effective: Jaundice was improved, and TBIL decreased by 10% to 30% on the fifth day after the operation. Ineffective: There was no improvement in jaundice, no change in TBIL, or a decrease of less than 10% on the fifth postoperative day. The total effective rate = (number of significantly effective cases + number of effective cases)/total number of cases × 100%. (4) Liver function indicators: Including TBIL, DBIL, and ALT. (5) Postoperative complications: Including postoperative bleeding, poor biliary drainage, biliary tract infection, acute pancreatitis, and so on. Two reviewers independently evaluated the quality of the included literature and extracted the data. In case of differing opinions, the decision on inclusion or exclusion was made in consultation with a third researcher.

### 2.4 Quality assessment

Methodological quality assessment of included studies: The Cochrane Collaboration’s Risk of Bias Assessment Tool was used to evaluate the quality, including: (1) random sequence generation; (2) allocation concealment; (3) blinding of participants and personnel, and blinding of outcome assessment; (4) incomplete outcome data; (5) selective reporting; (6) other sources of bias. In case of disagreements, the possibility of inclusion was discussed with the third researcher.

### 2.5 Statistical methods

Data were analyzed using RevMan 5.3 software. For binary response data, odds ratio (OR) was used to calculate 95% confidence intervals (Cl), while mean difference (MD) was used for continuous data. The I^2^ statistic and Q-test were employed to test the heterogeneity between the included studies. When P>0.10 and I^2^<50%, it indicates that the heterogeneity between the results of each study is small, and a fixed-effects model can be used to analyze the results; when P ≤ 0.10 and I^2^≥50%, it indicates that the heterogeneity between the results of each study is large. Sensitivity analyses were then conducted by excluding the literature one by one to re-examine the effect sizes, and the articles that influenced the results were further analyzed to identify the sources of heterogeneity. A random effects model was also used for the analysis. Possible publication bias was assessed using a funnel plot for outcome metrics with a number of included articles >10. A value of P < 0.05 was considered statistically significant.

## 3 Results

### 3.1 Study selection and characteristics of literature

**Figure 1:**
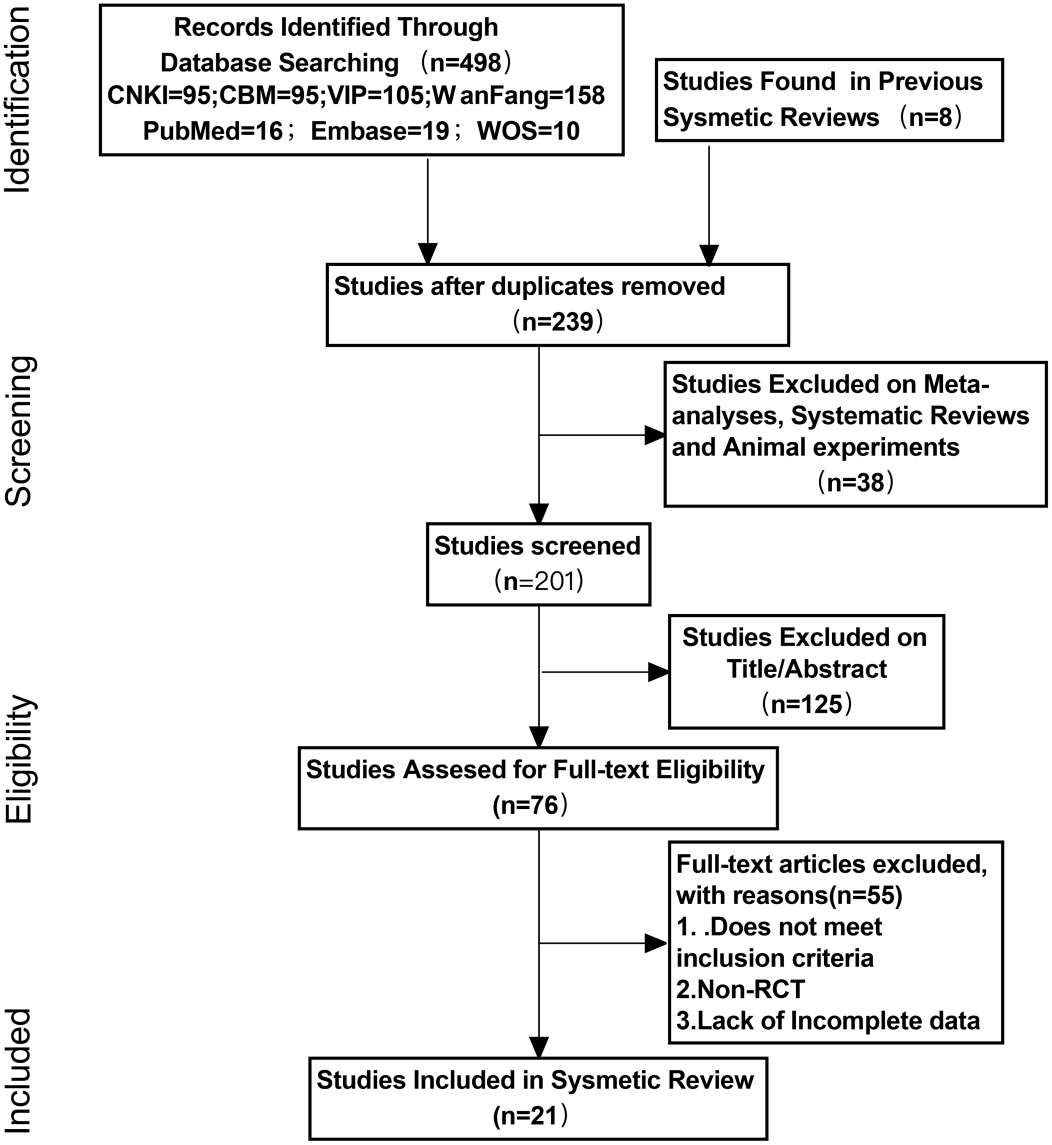
PRISMA flow diagram.

**Figure 2:**
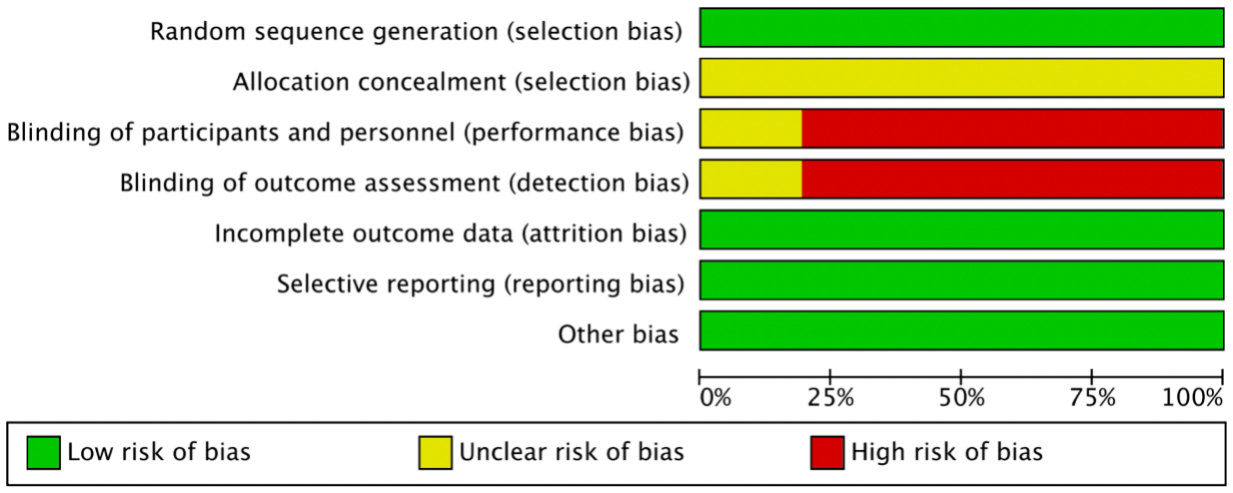
Quality assessment of the enrolled studies.

A total of 506 studies were retrieved using the two search strategies. The two researchers carefully reviewed the titles, abstracts, and full texts according to the inclusion and exclusion criteria, and conducted initial and secondary screenings. Finally, 21 studies (9–29) were included in the meta-analysis. The detailed screening process is illustrated in **Figure 1**. The basic characteristics of the included literature are presented in **Table 1**. The quality assessment of the included literature is shown in **Figure 2**.

**Table 1:** Basic characteristics of the included literature.

Author	year	Country	Design	No. Patients in study		study period	Outcome
				PTCD	ERCP		
Che JJ (9)	2019	China	RCT	45	45	2016-2018	②⑤
Chen ZS (10)	2019	China	RCT	25	18	2016-2018	②④⑤
Chi CK (11)	2019	China	RCT	39	39	2014-2017	①③④⑤
Coelen RJS (12)	2018	Netherlands	RCT	27	27	2013-2016	①③⑤
Ei-Haddad (13)	2021	Egypt	RCT	30	34	2019-2020	①③④⑤
He RH (14)	2020	China	RCT	30	30	2015-2019	②④⑤
Huang T (15)	2023	China	RCT	60	60	2019-2021	②④⑤
Huang YT (16)	2020	China	RCT	42	42	2017-2019	②④⑤
Li YG (17)	2020	China	RCT	48	48	2017-2019	③④⑤
Liu Y (18)	2016	China	RCT	50	50	2012-2015	③
Liu ZJ (19)	2017	China	RCT	44	44	2013-2016	①③⑤
Ma HY (20)	2017	China	RCT	47	50	2014-2015	①③④⑤
Pinol V (21)	2002	Spain	RCT	28	26	1996-1999	①③⑤
Saluja SS (22)	2008	India	RCT	27	27	NR	①③⑤
Sun XR (23)	2014	China	RCT	57	55	2006-2010	①③⑤
Wang CY (24)	2018	China	RCT	45	45	2012-2017	①②⑤
Wang Y (25)	2018	China	RCT	48	48	2015-2017	①②⑤
Wang YB (26)	2011	China	RCT	18	27	2007-2010	①④
Xu Z (27)	2019	China	RCT	30	30	2014-2017	④⑤
Zuo GZ (28)	2018	China	RCT	34	34	2016-2018	①
Zhou HB (29)	2019	China	RCT	70	70	2013-2016	③

① Surgical success rate; ② jaundice remission rate; ③ clinical effectiveness; ④ liver Function Indicators; ⑤ Overall complication rate.

### 3.2 Comparison between PTCD and ERCP

#### 3.2.1 Surgical success rate

**Figure 3:**
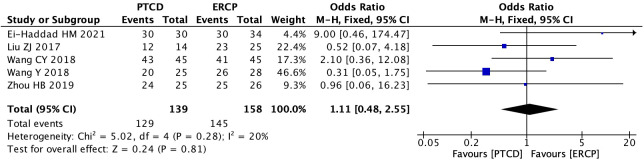
Forest plot of surgical success rate in patients with low obstruction.

**Figure 4:**
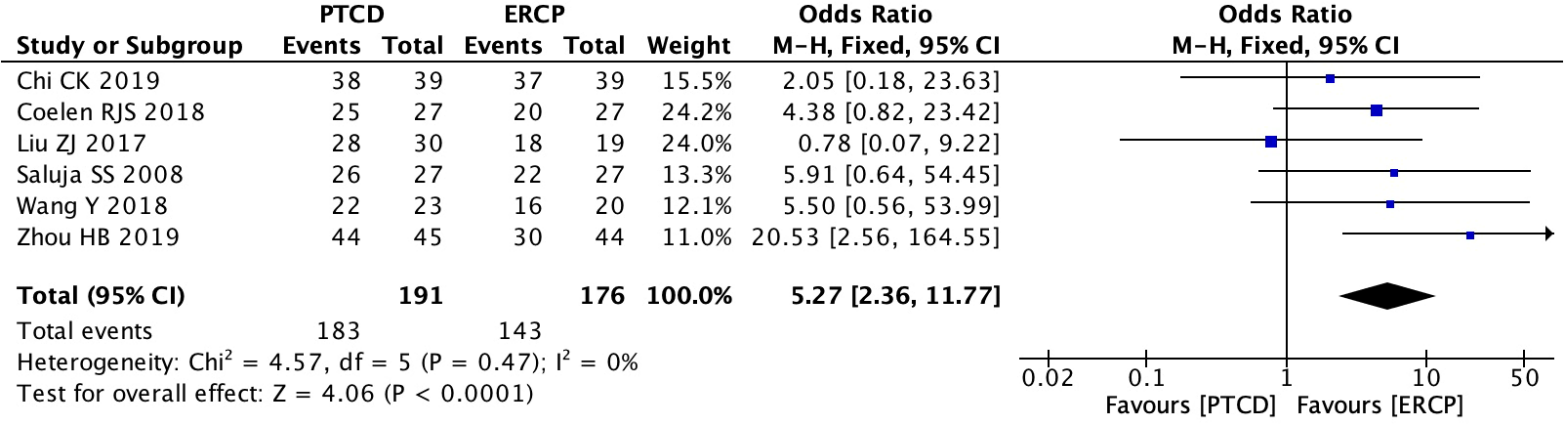
Forest plot of surgical success rate in patients with high obstruction.

**Figure 5:**
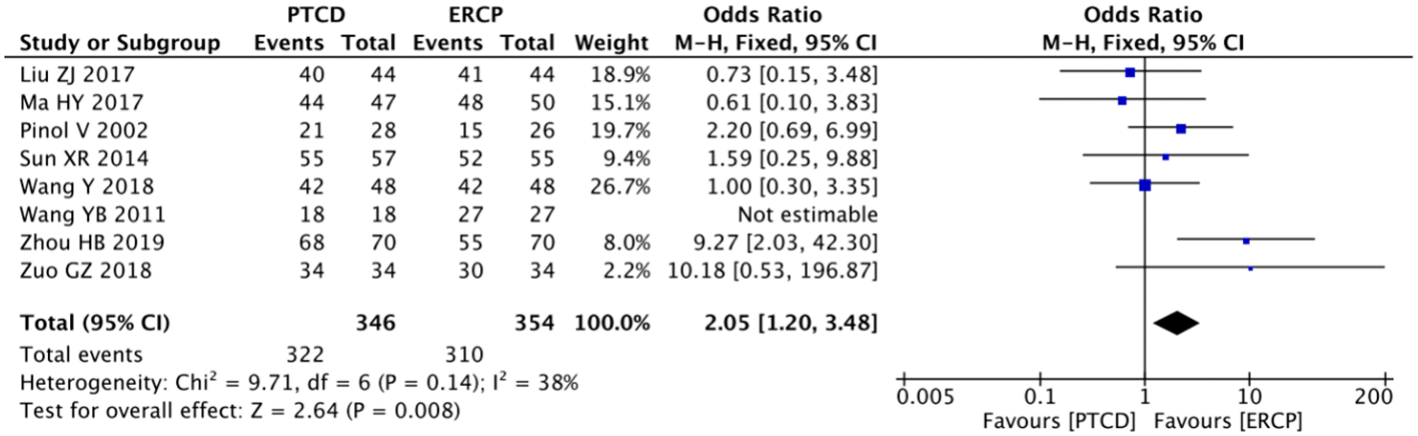
Forest plot comparing the overall surgical success rate.

Five studies (13, 19, 24, 25, 29) reported surgical success rates in patients with low-level obstruction, with low heterogeneity in outcomes across studies (P=0.28, I²=20%). Therefore, a fixed-effects model was used for data analysis, and the results of the meta-analysis were as follows: P=0.81, OR=1.11 (95% CI: 0.48–2.55). Statistically, the difference was not significant (**Figure 3**). Six studies (11, 12, 19, 22, 25, 29) reported surgical success rates in patients with high-grade obstructions, and there was no significant heterogeneity between the study results (P=0.47, I²=0%). Using the fixed- effects model, the results were as follows:P<0.0001, OR=5.27, (95% CI:2.36-11.77). The difference was statistically significant, indicating that the success rate of PTCD for high-level obstructions is higher (**Figure 4**). A total of 8 studies (19–21, 23, 25, 26, 28, 29) reported the overall surgical success rates, with relatively low heterogeneity between the study results (P=0.14, I²=38%). Therefore, the fixed-effects model was used to analyze the data, and the results were as follows: P=0.008, OR=2.05 (95% CI: 1.20–3.48). The difference was statistically significant, suggesting that the PTCD group had a significantly higher surgical success rate than the ERCP group (**Figure 5**).

#### 3.2.2 Jaundice remission rate

**Figure 6:**
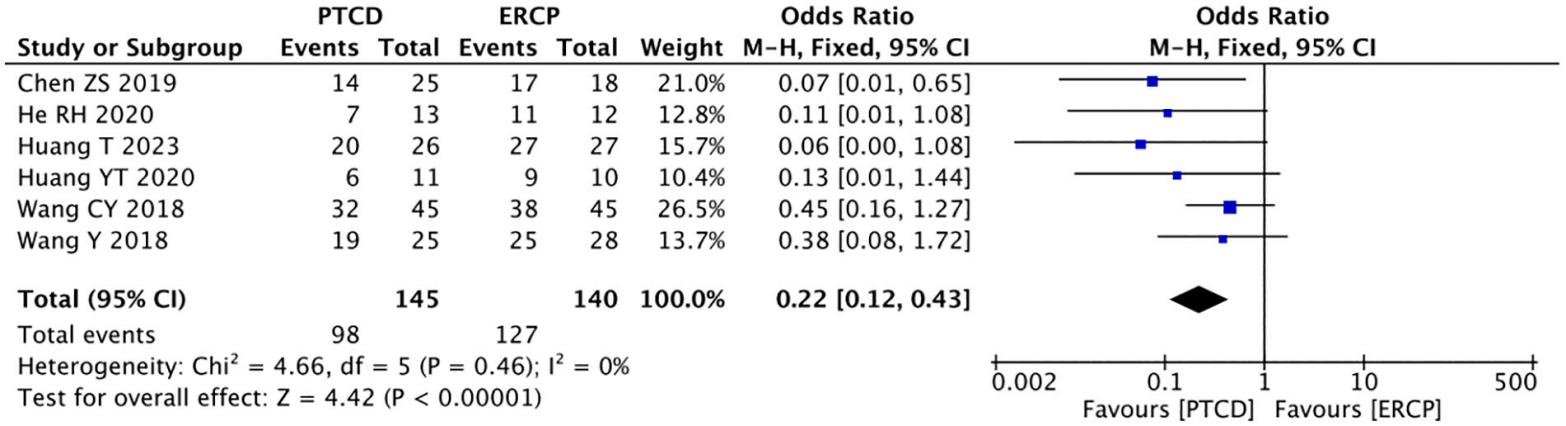
Forest plot of jaundice remission rate in patients with low obstruction.

**Figure 7:**
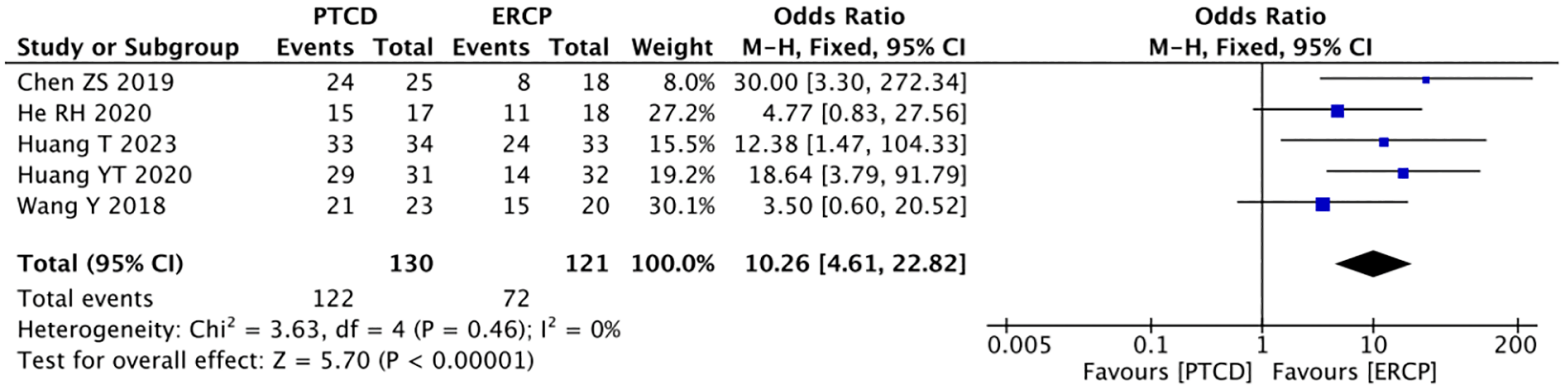
Forest plot of jaundice remission rate in patients with high obstruction.

Six studies (10, 14–16, 24, 25) reported the rate of relief of jaundice in low-level obstructions, and there was no significant heterogeneity among the study results (P=0.46, I²=0%).The data were analyzed using a fixed-effects model: p<0.00001, OR=0.22(95% CI: 0.12-0.43), The difference was statistically significant, indicating that in patients with low-level obstructions, ERCP leads to a more significant reduction in jaundice (**Figure 6**). Five studies (10, 14–16, 25) reported the jaundice remission rate in patients with high-grade obstructions, and there was no heterogeneity in the outcomes (P=0.66, I²=0%).Using the fixed-effects model: p<0.00001, OR=10.26(95%CI:4.61-22.82). The difference was statistically significant, and the jaundice remission rate are higher with PTCD in patients with high levels of obstructions (**Figure 7**).

**Figure 8:**
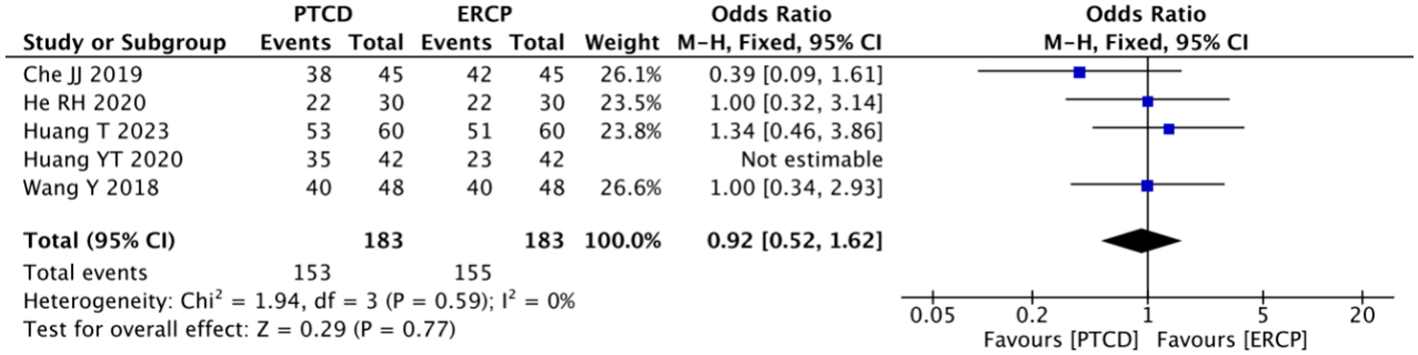
Forest plot comparing the overall jaundice remission rate.

The overall jaundice remission rate was reported in five studies (9, 14–16, 25), and heterogeneity was observed among the results (P=0.08, I²=51%). A sensitivity analysis was conducted, and it was found that the heterogeneity was significantly reduced after excluding the literature by Huang YT (16) (P = 0.59, I² =0%). Therefore, data analysis was performed using the fixed-effects model: p=0.77, OR=0.92 (95% CI: 0.52-1.62). The results were not statistically significant (**Figure 8**).

#### 3.2.3 Clinical effectiveness

**Figure 9:**
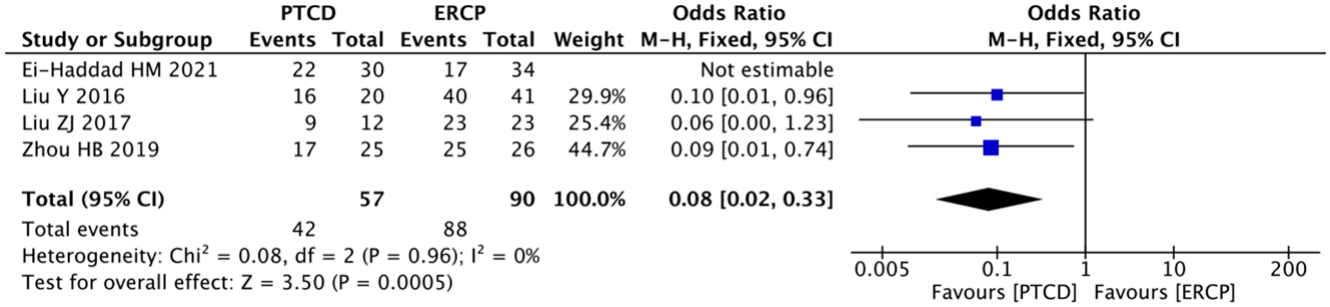
Forest plot of clinical efficacy in patients with low obstruction.

**Figure 10:**
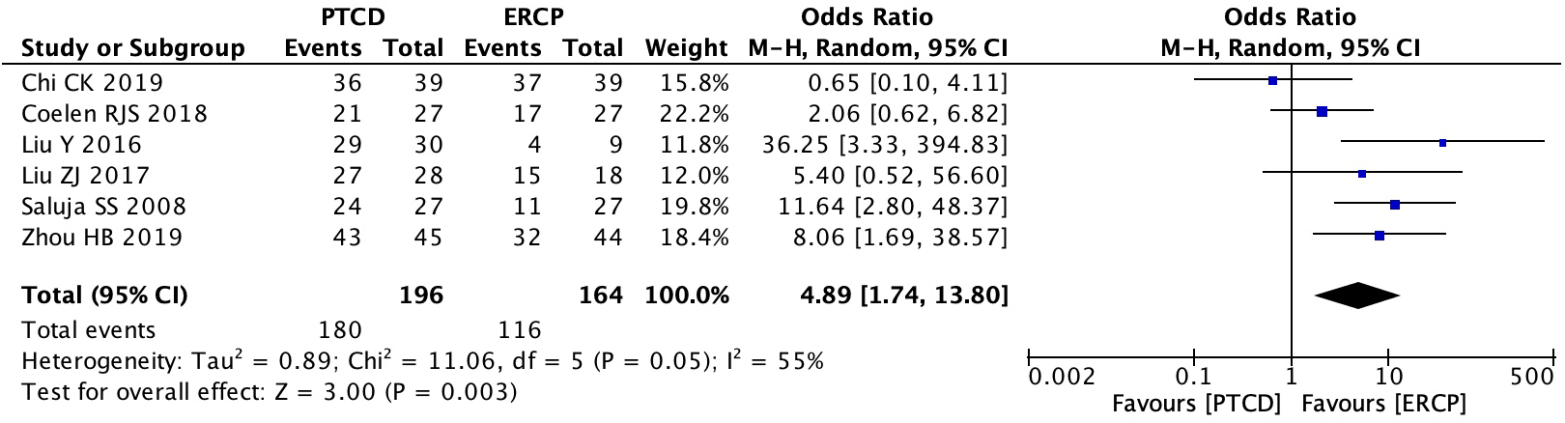
Forest plot of clinical efficacy in patients with high obstruction.

**Figure 11:**
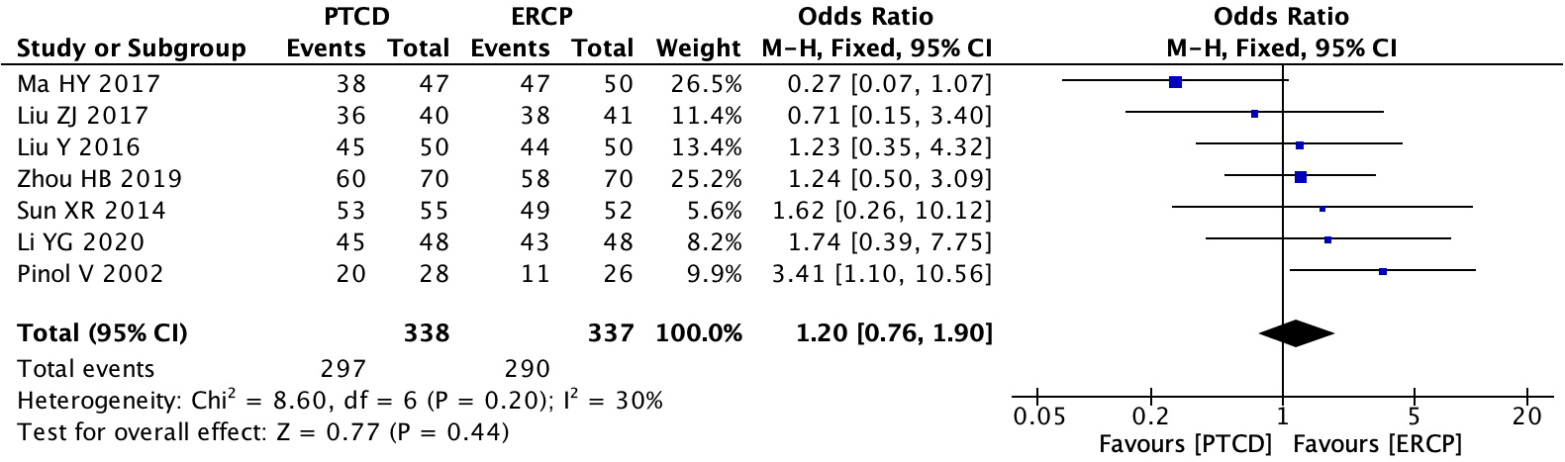
Forest plot comparing the overall complication rate.

Four articles (13, 18, 19, 29) described the clinical efficacy of patients with low obstruction, and there was a large heterogeneity among the results of each study (P=0.001, I²=81%). A sensitivity analysis was performed on these articles, and after excluding the study of Ei-Haddad HM (13), there was a significant decrease in heterogeneity (P=0.96, I²=0%).The fixed effect mode was applied, and the results of the meta-analysis were as follows: P=0.0005, OR=0.08(95% CI: 0.02-0.33). The difference was statistically significant, indicating that patients with low-level obstructions had better clinical outcomes when treated with ERCP (**Figure 9**). The clinical outcomes of patients with high-grade obstructions were reported in six studies (11, 12, 18, 19, 22, 29), with significant heterogeneity among the studies (P=0.05, I²=55%). A sensitivity analysis was conducted, and it was found that there was no study with a significant impact on heterogeneity. The random effect model was used, and the results were as follows:P=0.003, OR=4.89(95% CI:1.74~13.80), suggesting that PTCD is more effective in treating high-level obstructions (**Figure 10**). The overall clinical efficacy was reported in seven studies (17–21, 23, 29), and there was little heterogeneity among the results (P=0.20, I²=30%). Therefore, a fixed-effects model was used for data analysis, and the results were as follows: P=0.44, OR=1.20(95% CI:0.76-1.90). There was no statistical difference between the two procedures (**Figure 11**).

#### 3.2.4 Liver function indicators

A total of seven studies (10, 14, 16, 17, 20, 26, 27), nine studies (10, 11, 13–17, 20, 26), and eight studies (10, 11, 13–17, 20) reported the pre-surgical and post-surgical differences in ALT, TBIL, and DBIL, respectively, and there was significant heterogeneity in the findings (I²=73%, 91%, 88%). A sensitivity analysis was performed, and it was found that the change in heterogeneity was not significant before and after excluding the literature one by one. Therefore, the random effects model was used, and the results were as follows: P=0.93, 0.09 and 0.55. All differences were not statistically significant (**Table 2**).

**Table 2:** Comparison of the decreased of ALT, TBIL and DBIL between the two groups.

Liver function indicators	Z	P	OR	95%Cl	Heterogeneity	
					P	I²
ALT	0.09	0.93	-0.19	-4.16-3.79	0.0009	73%
TBIL	1.67	0.09	-5.67	-12.31-0.97	<0.00001	91%
DBIL	0.60	0.55	-1.77	-7.54-4.00	<0.00001	88%

#### 3.2.5 Overall complication rate

**Figure 12:**
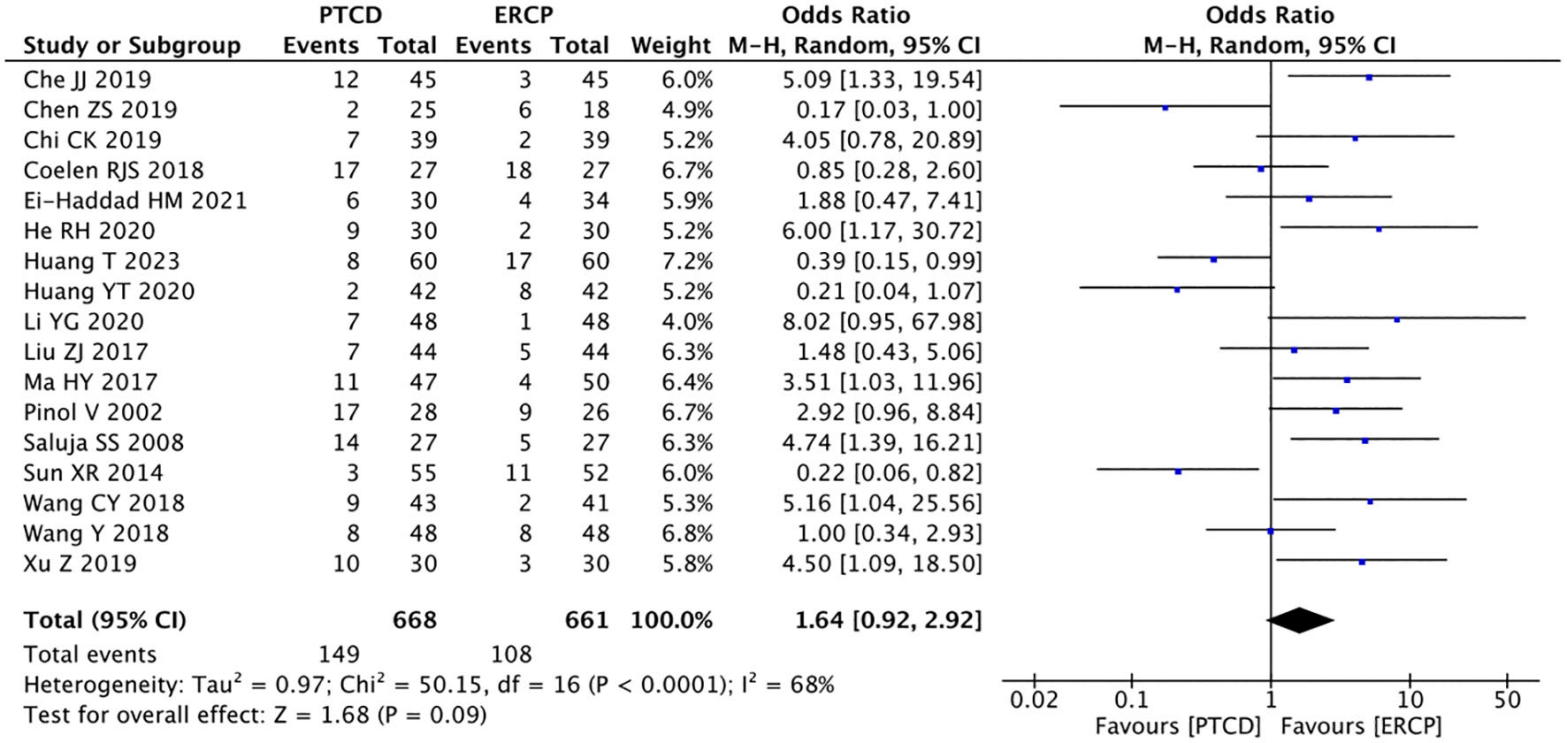
Forest plot of the overall complication rate.

A total of 17 studies (9–25, 27) reported the incidence of postoperative complications, and the heterogeneity among the studies was considerable (P<0.001, I²=68%). Sensitivity analysis revealed that no single study had a significant impact on the heterogeneity. Employing the random effects model: P = 0.09, OR = 1.64(95%CI:0.92-2.92), the overall complication rate in the PTCD group was 1.64 times higher than that in the ERCP group, however, the difference was not statistically significant. This indicates that there was no significant difference in the complication rate between the PTCD and ERCP groups (**Figure 12**).

### 3.3 Publication bias

**Figure 13:**
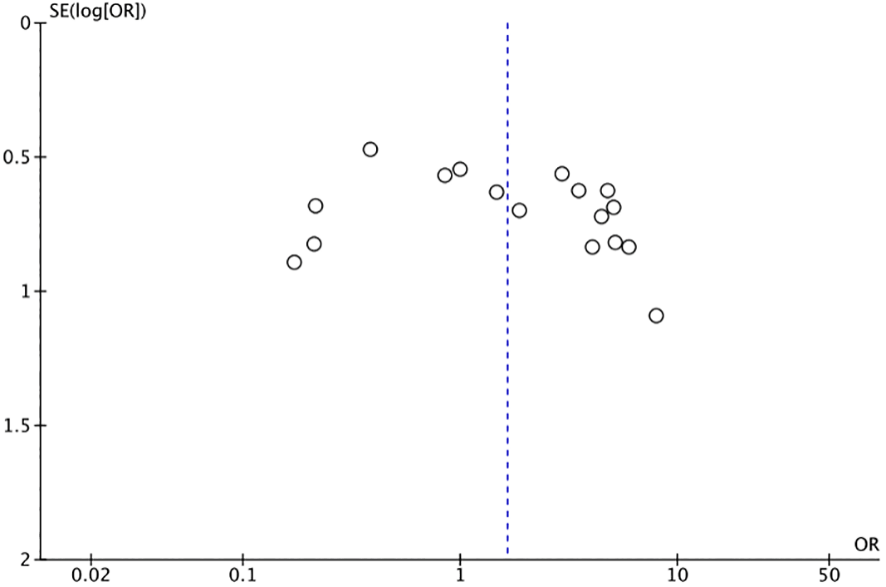
Funnel plot of the rate of complication.

**Figure 14:**
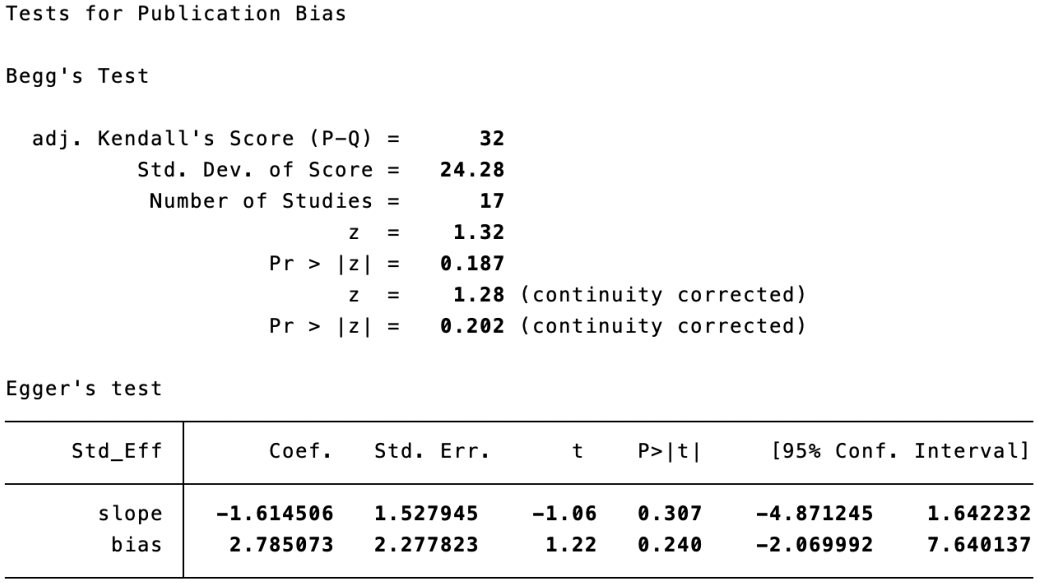
Begg’s test and Egger’s test of the rate of complication.

Based on the funnel plot of the complication rate, a publication bias analysis was conducted. Additionally, the Egger’s test was performed, and the results indicated the absence of publication bias (**Figures 13**, **14**).

## 4 Discussion

Malignant obstructive jaundice is caused by direct or indirect stricture or occlusion of intrahepatic and extrahepatic bile ducts due to malignant tumors, resulting in obstruction of bile excretion and stasis, which can lead to a series of serious complications such as hyperbilirubinemia, coagulation dysfunction, liver and kidney dysfunction (30, 31). The common causes of MOJ include primary bile duct cancer, gallbladder cancer, liver cancer, pancreatic cancer and periampullary cancer, which cause metastasis or invasion of the bile ducts. Due to its high degree of malignancy, it can pose a threat to the life of patients if not treated in a timely manner. The related treatment is usually palliative, and the main purpose of treatment is to improve the liver function of patients (32). Currently, different studies have demonstrated that both PTCD and ERCP are effective and safe.

In this study, a total of 21 RCTs were selected through two search pathways to systematically evaluate the efficacy and safety of PTCD and ERCP in the treatment of MOJ. When reviewing the included literature of similar related Meta-analyses published in the past, it was discovered that in the study of GH Bao et al. (33), which had been included in previous Meta-analyses, the specific grouping principles were not mentioned. Instead, it was simply divided into two groups based on the treatment method or the patients’ voluntary choice of surgery, raising suspicions of a grouping bias caused by the patients’ knowledge background or other factors. The possibility of nonrandomization was considered to be high. Therefore, it did not meet the principle of inclusion of randomized controlled trials in this study and was not included.

The comparison of efficacy in this study indicates that: (1) There is no significant difference in the surgical success rate between the two procedures in patients with low-level obstruction, but the overall surgical success rate and the success rate in patients with high-level obstruction via PTCD are higher. This may be due to the fact that, compared to ERCP, PTCD requires direct puncture of the hepatic parenchyma, resulting in a shorter path to the site of the obstruction. The influencing factors of PTCD include: whether the patient can tolerate the operation, whether the guide wire can successfully pass through the bile duct stenosis, and the number of stents placed. The influencing factors of ERCP include the ability to successfully identify the duodenal papilla endoscopically, the ability of the guide wire to successfully pass through the biliary stricture, and the skill of the operator (34). Some studies have also reported a failure rate of up to 10% for ERCP procedures,and PTCD is often used as an alternative in cases of ERCP failure (35). (2) There was no significant difference in the overall jaundice remission rate and clinical efficacy between PTCD and ERCP, but depending on the site of obstruction, ERCP maneuvers were more effective in patients with low obstructions, while PTCD was better in jaundice remission in patients with high obstructions. (3) There was no significant difference in ALT, TBIL and DBIL before and after operation between the two groups. In conclusion, the success rate of PTCD is higher than that of ERCP, and it can be recommended as the first choice of treatment or as a remedy after the failure of ERCP treatment. Both surgical procedures have a certain efficacy in reliving jaundice and improving liver function. In practice, we can analyze the specific conditions of the patients and try to choose the most reasonable treatment plan.

Any interventional procedure is invasive, and the efficacy is accompanied by the risk of complications. Both procedures may lead to a number of complications. The main complications of PTCD are bleeding and biliary tract infection. The main cause of bleeding is the obscuration of the puncture path of PTCD and the inadvertent puncture of the patient’s blood vessels during the puncture. The bleeding is caused by the blinding of the puncture path of PTCD, and inadvertent puncture of the patient’s blood vessels during the puncture (36). The infection may be secondary to the reflux of duodenal fluid or to an associated poor drainage process, while ERCP is more likely to cause acute pancreatitis (37). Some scholars have also reported that the incidence of postoperative complication rates is as high as 30% to 50% for PTCD, compared to only about 5% for ERCP (38). The results of the meta-analysis showed that although the overall complication rate was higher in the PTCD group than in the ERCP group, it was not statistically significant, which is inconsistent with the results of some previous studies (39, 40). It may be due to the uneven grouping of some studies, which led to a bias towards patients with high levels of obstruction. These patients have greater difficulty in performing ERCP, thus increasing the complication rate in the ERCP group. However, the results of these studies may also be related to geographic areas, target populations, sample sizes, and other factors.

The shortcomings of this study are as follows: Through the search and screening, only a limited number of literatures were included, and fewer relevant RCT studies from abroad were retrieved that met the criteria. In addition, the inclusion of literature did not specify the blinding and allocation concealment, which may be subject to bias.

In summary, this study indicates that both ERCP and PTCD have comparable clinical effectiveness in treating MOJ, as they can both effectively alleviate jaundice and reduce biliary obstruction. The overall success rate of PTCD is higher than that of ERCP, while the incidence of postoperative complications is slightly lower. A more suitable treatment option can be chosen based on the location of the obstruction. PTCD shows better clinical outcomes in patients with high obstruction, while ERCP performs better in those with low obstruction.

## Data availability statement

The datasets presented in this study can be found in online repositories. The names of the repository/repositories and accession number(s) can be found in the article/**Supplementary Material**.

## Author contributions

YW: Conceptualization, Methodology, Data analysis, Writing – original draft, Writing – review & editing. XC: Conceptualization, Methodology, Writing – review & editing. XZ: Writing – original draft, Writing – review & editing. YS: Writing – original draft. QK: Writing – original draft.
