# Supplementary material for: The clinical efficacy and safety of different biliary drainage in malignant obstructive jaundice: a meta-analysis
Source: Front Oncol. 2024 Apr 9;14:1370383. doi: 10.3389/fonc.2024.1370383 (PMC11035768; doi:10.3389/fonc.2024.1370383)
Supplement: Supplementary file 1 [file Table_1.docx]

Q20

APPENDIX

TABLE A1 PubMed Literature Search Strings

| PubMed was searched using the following terms:  (((Malignant Obstructive Jaundice) OR (MOJ[Title/Abstract])) AND ((Percutaneous transhepatic cholangial drainage) OR (PTCD[Title/Abstract]))) AND ((Cholangiopancreatography, Endoscopic Retrograde) OR ((((((Cholangiopancreatography, Endoscopic Retrograde[Title/Abstract]) OR (Cholangiopancreatographies, Endoscopic Retrograde[Title/Abstract])) OR (Endoscopic Retrograde Cholangiopancreatographies[Title/Abstract])) OR  (Retrograde Cholangiopancreatographies, Endoscopic[Title/Abstract])) OR (Endoscopic Retrograde Cholangiopancreatography[Title/Abstract])) OR  (ERCP[Title/Abstract]))) |
| --- |
